# The effect of titanium-platelet rich fibrin on periodontal intrabony defects: A randomized controlled split-mouth clinical study

**DOI:** 10.1371/journal.pone.0304970

**Published:** 2024-06-06

**Authors:** Didem Ozkal Eminoglu, Taner Arabaci, Gurbet Alev Oztas Sahiner

**Affiliations:** 1 Department of Periodontology, Faculty of Dentistry, Atatürk University, Erzurum, Turkey; 2 Department of Periodontology, Erzurum Oral and Dental Health Center, Erzurum, Turkey; University of Catania: Universita degli Studi di Catania, ITALY

## Abstract

This study aimed to determine the contribution of titanium prepared platelet-rich fibrin (T-PRF) with open flap debridement (OFD) on clinical, biochemical and radiographic measurements of periodontal regeneration. Twenty periodontitis patients with bilateral intrabony defects and stage III grade A periodontitis were included in the study. A total of 40 defects were randomly selected for OFD alone (control group, n = 20) or combined OFD+ T-PRF (test group, n = 20). Clinical and radiographic parameters (at baseline and nine months after surgery), and growth factor levels in gingival crevicular fluid (at baseline and at two, four, six, and twelve weeks after surgical treatment) were also evaluated. Considering the clinical parameters, alterations in probing pocket depth, gingival marginal level and clinical endpoint in the test regions treated with T-PRF significantly improved (*P*<0.05). Fibroblast growth factor-2 and platelet-derived growth factor-BB levels between the two groups in the second and fourth weeks were also significantly different (P<0.05). Furthermore, the receptor activator of nuclear factor κB ligand/osteoprotegerin ratio between the groups was significantly different in the second, fourth, sixth, and twelfth weeks (P<0.05). The bone-filling rate was also significantly greater in the test group than in the control group (P <0.001). Compared with OFD alone, combining T-PRF with the procedure was more successful with regards to clinical, radiographic, and biochemical measurements of periodontal regeneration.

## Introduction

Periodontitis is a multifactorial inflammatory disease that destroys the soft and hard tissues surrounding a tooth [1]. A common goal of any periodontal treatment is improving this inflammation, reducing the probing pocket depth (PPD), and increasing the clinical attachment level (CAL). Treating intraosseous defects and regenerating lost hard tissue is challenging in periodontology, and the effectiveness of nonsurgical and surgical periodontal treatments is evaluated based on the positive effects on bone levels [2]. When treating periodontal defects, current practice includes open flap debridement (OFD) either alone or in combination with autografts, allografts, xenografts, synthetic grafts, platelet-derived growth factor, or fibroblast growth factor to enhance bone growth [3]. Although OFDs provide gains in soft tissues, they do not have such an effect on hard tissue [4].

Platelet concentrates are important biomaterials that aid in tissue healing and periodontal regeneration by stimulating the surrounding progenitor cells [5]. These cells secrete growth factors, fibronectin, bone morphogenetic proteins, and cytokines over specific periods of time [6]. Initially, platelet-rich plasma (PRP), the first form of platelet concentrates, was used alone or in combination with bone grafts to treat intraosseous defects. Although the treatment results were satisfactory, there was a risk of antigenicity due to adding bovine antithrombin to PRP. For this reason, leukocyte-platelet-rich fibrin (L-PRF), obtained without the need for any anticoagulant, was introduced in 2001 by Choukroun et al. [7]. Since then, L-PRF has been actively used in periodontal regeneration, and its success has been demonstrated clinically and radiographically [8–10]; however, it has potential limitations including cross-contamination with silica, a lack of rigidity, and fast degradation [10]. A review by Miron et al. [11] revealed the harmful effects of tubes containing silica and silicone. The same study reported that silica contained in tubes led to a significant reduction in PRF clot size. Tsujino et al. [12] recommend using silica-containing tubes only once their safety was guaranteed. They reported that between 5% and 30% of silica microparticles were directly incorporated into the PRF matrix and indirectly into oral tissues via PRF clots. Tsujino et al. [12] also found that silica microparticles were adsorbed onto cell surfaces, causing cell apoptosis and reducing cell proliferation and viability. Given the cross-contamination of L-PRF with silica, O’Connell [13] and Tunalı et al. [14] investigated a new biomaterial that did not use glass or glass-coated vacutainer tubes. They produced a new biomaterial in a titanium tube because of its osseointegration, better biocompatibility, and ability to activate platelets. A third-generation platelet concentrate called titanium-prepared platelet-rich PRF (T-PRF) results in the formation of a thicker fibrin layer, a longer absorption time, increased capacity for osseointegration, better hemocompatibility, greater cellular support, and it promotes periodontal regeneration [15]. Moreover, T-PRF is resorbed later in the applied area than L-PRF, allowing for more growth factor release than L-PRF [14, 16, 17].

Miron et al. [18] stated that for the quality of content and size for PRF clots, the selection of tubes is more important than centrifugation device or centrifugation speeds. Therefore, this study aimed to determine the contribution of T-PRF with OFD on the clinical, biochemical, and radiographic measurements of periodontal regeneration. We hypothesized that an OFD operation combined with T-PRF would positively affect clinical success and biochemical markers.

## Materials and methods

### Experimental design

In this study, different modalities for treating deep periodontal intrabony defects (IBDs) were compared. This was a split-mouth (two different and independent sites within the mouth of a single patient), randomized (in which the site of the jaw treated with any particular method was determined randomly), parallel clinical study. Three-wall defects in a control group were treated with OFD only, while the 3-wall defects in the test group were treated with OFD supplemented with T-PRF. The same periodontal treatment procedure was applied in both groups, except for the use of T-PRF. Clinical and radiographic parameters were measured from baseline to nine months after surgery.

### Sample size calculation

The sample size was calculated based on the hypothesis ‘H0: There is no statistically significant difference between the mean values of probing depths between OFD and OFD+T-PRF treated groups.’ The article by Kizildağ et al. [19] was used to determine the sample size. We calculated that 16 jaw sites should be used in each group to achieve 80% power and a 95% confidence level for a difference of 2 ±2 mm units between the probing depth averages in the experimental and control groups. Considering potential losses, 20 sites were included in each group.

### Selection of participants

This split-mouth, randomized, controlled clinical trial was approved by the Medical Faculty Non-Pharmaceutical Clinical Research Ethics Committee of XXX University (Protocol Code of The Research: B30.2.ATA.0.01.00/01; meeting: 25.03.2021/2; decision no: 1; Reference Number: B30.2.ATA.0.01.00/93). The study protocol for the clinical trial approved by institutional review board (IRB) was registered, and posted on the ClinicalTrials.gov public website (ClinicalTrials.gov Identifier: NCT05409495) (S1 File). The study was conducted in accordance with the World Medical Association Declaration of Helsinki, last revised in 2013. Oral and written information about the study was given to all potential study participants. Informed consent was signed by the patients who agreed to participate.

The study population consisted of 20 participants (11 males and 9 females) aged >20 to 60 years who attended the outpatient department of Periodontics, Faculty of Dentistry, XXX University, Erzurum, TURKEY. The participants were identified through the evaluation of 55 patients. The study was conducted from April 2021 to May 2022. Patients diagnosed with stage III, grade A periodontitis. Inclusion criteria were: patients with interdental CAL ≥5 mm extending to the middle third of the root and beyond, radiographic bone loss, tooth loss (≤4) due to periodontitis, nonsmokers, and systemically healthy individuals. Individuals were further selected according to the criteria of the American Academy of Periodontology (2017) classification system of periodontal diseases and included in the study [20]. Participants consisted of patients with bilaterally similar periodontal 3-wall IBDs who were clinically and radiologically found to have a probing depth ≥5.0 mm after nonsurgical periodontal treatment [21].

The following patients were excluded: individuals who did not follow the necessary oral hygiene instructions during the nonsurgical periodontal treatment process; those with a history of periodontal therapy in the preceding year; the presence of a devital tooth, Grade II [22] high tooth mobility; less than three bone walls or a defect in the furcation at the site of the bone defect, the presence of marginal tissue recession, gingival enlargement, pseudopockets or combined pockets; a history of any systemic disease that could alter the course of periodontal disease; smokers; the use of antibiotics; and pregnant or lactating women.

**Fig 1:**
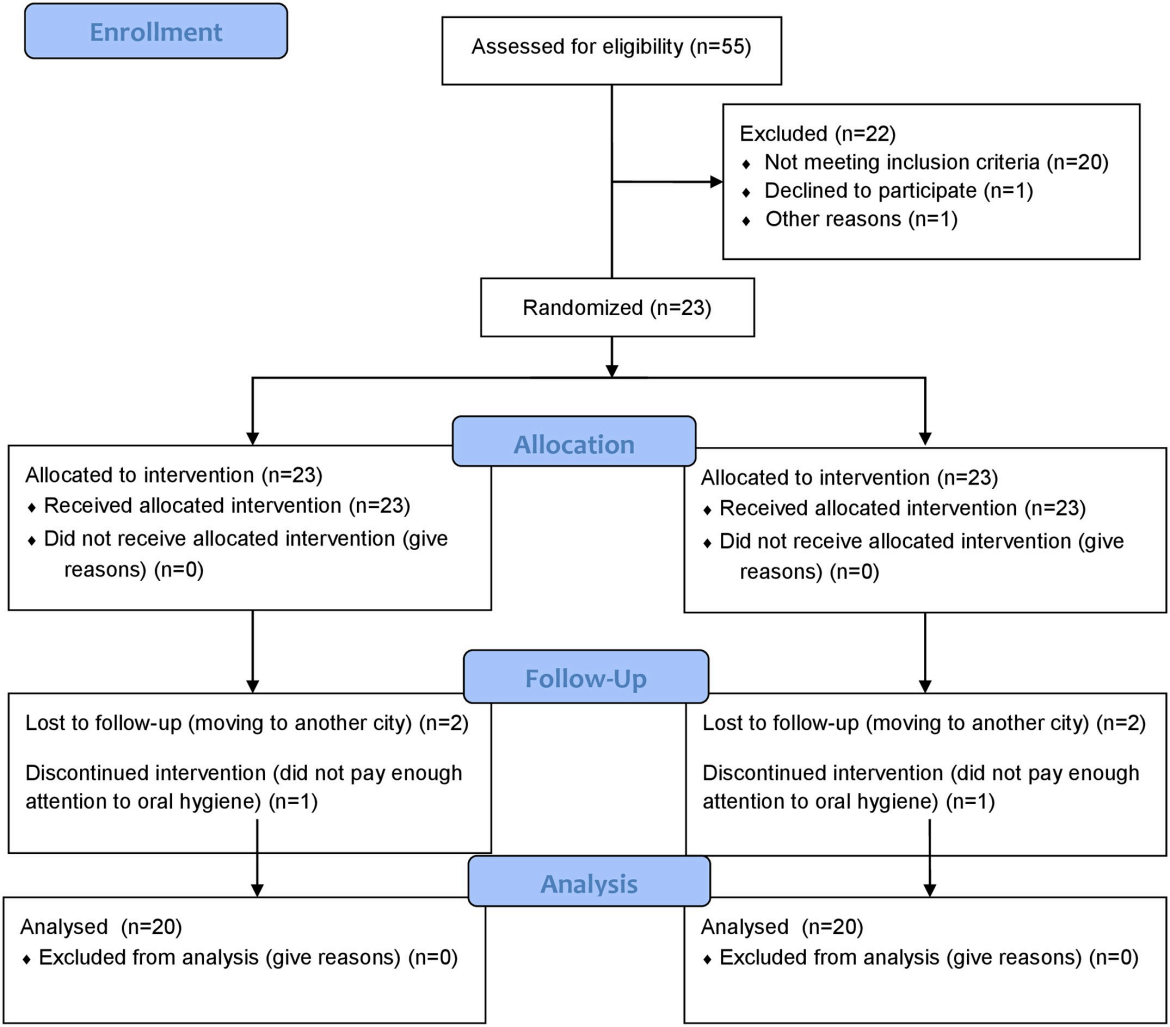
Flow diagram for the split-mouth, randomized controlled clinical trial.

One of the investigators (TA) identified the participants by evaluating 55 patients. Twenty-two were excluded at baseline, and 23 were allocated to the intervention arm. During the follow-up visits, two patients were lost to follow-up. One patient was excluded from the study because they did not follow the post-surgical oral hygiene instructions (Fig 1).

### Clinical and radiographic measurements

One investigator (GAÖŞ, a periodontist) was appointed to perform the initial and postoperative (nine months after surgery) clinical and radiographic measurements and collect gingival crevicular fluid (GCF) samples. The periodontist was blinded to the allocation of the patients (experimental or control) when taking measurements. Periodontal parameters were recorded using a Williams periodontal probe (Hu-Friedy, Chicago, IL, USA). The periodontal parameters included the site-specific plaque index (PI) (Silness & Loe) [23], the modified sulcus bleeding index (mSBI) [24], PPD (evaluated from the gingival margin to the base of the pocket) [25], the gingival marginal level (GML) (measured from the most apical end of the stent to the crest of the gingival margin) [10], and the CAL (evaluated from the cemento-enamel junction to the base of the pocket and GML) [26]. To serve as a fixed reference point for the measurements, PPD, GML, and CAL were measured in the defect area using customized acrylic stents and trimmed to the height contour of the teeth [27]. Intra-examiner calibration was performed by evaluating the measurements of 10 patients 24 hours apart prior to the start of the study. Calibration was considered only if the baseline and 24-hour scores were within ±1 mm at the 90% level [28].

When calculating the PI, four surfaces of each tooth were evaluated. The absence of plaque (0), the presence of plaque after probing along the gingival margin (1), the presence of visible plaque (2), and the presence of dense plaque (3) were counted. A tooth score was calculated by dividing the sum of the scores of the surfaces by four, and an individual score was calculated by dividing the sum of the tooth scores by the total number of teeth. When calculating the mSBI, bleeding states occurring within 15 seconds after probing were scored as 1. The individual’s score was calculated by dividing the dental score by the number of existing teeth. Four surfaces of each tooth were evaluated when calculating the PPD, GML, and CAL. The surface with the highest probing depth was determined as the tooth score.

Radiographic images were recorded at baseline and nine months after surgery. An individually customized bite block, a film holder device, and a parallel angle technique were used to standardize the participants’ radiographic recordings. While examining a radiographic intraosseous defect, the distance between the top of the alveolar bone crest (A) and the deepest point of the defect (B) was measured. The A-B distance was evaluated using computer-aided software (Scion Image Analyzer, Scion, Frederick, MD) [29].

When measuring periodontal bone support (PBS) using radiographic images, Image Tool v.3.0 (UTHSCSA; Informer Technologies Inc., Los Angeles, CA, USA) was used. Measurements were made at baseline and after the surgical procedure to evaluate bone acquisition in the defect area. All measurements were made with three reference points located distal to the mandibular first molars (the apex of the distal root (A), the apical level of the defect located in the distal-apical part of the tooth (B), and the distal cusp tip (C)). The apex-deepest bone defect (AB) and the apex-cusp tip (AC) distances were measured in mm. The PBS concentration was calculated using the formula PBS = AB/AC ×100 [30].

### Gingival crevicular fluid collection

GCF was collected via the intracrevicular technique [31] using paper strips at the gingival groove to evaluate fibroblast growth factor-2 (FGF-2) and platelet-derived growth factor-BB (PDGF-BB) levels and the relative ratio of receptor activator nuclear factor kappa-B (RANKL)/osteoprotegerin (OPG). GCF was collected at baseline and 2, 4, 6, and 12 weeks after surgical treatment. GCF was subdivided into superficial and deep, depending on the depth of strip insertions into the gingival sulcus or periodontal pocket. Absorbed GCF samples were evaluated with a calibrated instrument (Periotron 8000; Oraflow, Plainview, NY, USA). The samples were then placed in polypropylene tubes containing 500 μL of phosphate-buffered saline solution and stored at -80°C for later use.

### Randomization and allocation concealment

Before surgical procedures were begun, the periodontal intraosseous defects on the right and left sides of each patient were randomly divided into two groups according to the surgical procedure (by complete randomization): OFD alone and OFD + T-PRF with autologous T-PRF. The investigator (DÖE) performing the surgical procedures was blinded to information about the side of a patient’s jaw that was the control and experimental.

### Presurgical therapy

An investigator (DÖE) conducted oral hygiene education, Phase 1 periodontal therapy, and surgical periodontal therapy. It took approximately four months to complete the supra/subgingival debridement procedures and prepare the patients for the surgical phase.

### Preparation of T-PRF

Just before surgery, 20 mL of intravenous blood from the antecubital vein was collected in two titanium tubes without anticoagulant. Blood samples were immediately centrifuged for 10 min at 3,000 rpm at room temperature in a PC-02 centrifuge (Hettich^®^ EBA 20 centrifuge; Hettich GmbH & Co. KG, Tuttlingen, Germany). At the end of the centrifugation, three parts were obtained in the tube: red blood cells at the bottom, platelet-poor plasma at the top, and fibrin layers (the ‘buffy coat’) in the middle. Two fibrin layers were removed from the tubes using sterile tweezers and placed in a special PRF box (Professional German grade PRF and GRF Box System Platelet Rich Fibrin Dental Implant Surgery Instruments, Germany) to release the serum slowly over 20 minutes for surgery [14].

### Surgical procedure

Surgical procedures for the control and experimental groups (and for one participant with two bilateral defects) were performed by DÖE. A 0.12% chlorhexidine (CHX) digluconate rinse was used for 60 s to intraoral antisepsis, and a povidone-iodine solution was used for extraoral antisepsis. After local anesthesia (2% lidocaine with epinephrine 1:100,000/Astra, Westbrough, MA, USA) was applied, intracrevicular incisions were made using an 11-blade steel scalpel, and full thickness mucoperiosteal flaps were elevated enough to provide an adequate view of the defect area. In order to have vision for both the buccal and oral sides of the bone defects, a double flap technique was preferred [32]. Subgingival debridement and root planning were performed with the use of area-specific curets (gracey curets, Hu-Friedy), and granulation tissue was removed. The blood supply of the defect areas was taken into account. The IBD areas in the control group were closed without any material being applied. At the test site, IBDs were filled with one T-PRF, and the other T-PRF membrane was adapted over the defects both buccally and lingually, in addition to undergoing an OFD procedure. The mucoperiosteal flaps were then repositioned and sutured with 4/0 monofilament polypropylene sutures (4–0 Prolene^®^; Ethicon, Inc., Somerville, NJ, USA).

### Postoperative care

The patients were given antibiotics (875 mg amoxicillin and 125 mg clavulanic acid) (1000 mg tablets; GlaxoSmithKline, Istanbul, Turkey) twice a day for seven days, analgesics (550 mg naproxen sodium; Bilim Ilac, Istanbul, Turkey) twice a day for seven days, mouthwash (CHX digluconate rinses; 0.12%; Drogsan, Ankara, Turkey) twice a day for four weeks, and provided with a diet program [33]. Patients were informed that the drugs and mouthwashes should be taken at the appropriate dose and time intervals and were reminded to follow their oral hygiene instructions. Sutures were removed two weeks after surgery. The patients were examined two, four, six, twelve weeks, and nine months after surgery. Maintenance appointments were scheduled every month for the duration of the study for clinical evaluations and to reinforce oral hygiene instructions. Professional maintenance (gentle supragingival debridement) was performed at follow-up appointments—if necessary. Neither probing nor subgingival instrumentation was used until nine months after surgery. Surgical procedures were completed within two months. There were no cases of infection or other adverse complications at any treatment site throughout the study.

### Biochemical analyses

Specific enzyme-linked immunosorbent assay kits (ELISA; R&D Systems, Abingdon Science Park, Abingdon, UK) and a quantitative sandwich enzyme immunoassay technique were used to determine the relative PDGF-BB and FGF-2 levels and the RANKL/OPG ratio in the GCF samples. The kits were used according to the manufacturer’s instructions. Three samples from each patient were analyzed separately and interpreted using the standard curves of the kits.

### Statistical analyses

All statistical analyses were conducted using SPSS 20 software (IBM Corp, Armonk, NY, USA). Descriptive statistics were calculated, including the mean, standard deviation, percentage, and counts. The normality of the distribution of continuous variables was assessed using the Shapiro-Wilk test, the Kolmogorov-Smirnov test, Q‒Q plots, skewness, and kurtosis. For comparisons between two independent groups, the independent sample t-test was used when the normality of the distribution was met; otherwise, the Mann-Whitney U test was used. When comparing two dependent groups, the paired samples t-test was used if the normality of the distribution was satisfied; otherwise, the Wilcoxon test was used. For comparisons involving more than two dependent groups, repeated-measures ANOVA was performed under the assumption of a normal distribution; otherwise, the Friedman test was used. The assumption of sphericity was assessed in the repeated measures test, and either the sphericity assumption or the Greenhouse-Geisser method was used accordingly. Following the repeated measures test, post hoc tests were conducted using Tukey’s test for homogeneous variances and Tamhane’s T2 test for nonhomogeneous variances. After the Friedman test, post hoc tests were performed using Friedman’s 2-way ANOVA by ranks (k samples). For 2×2 comparisons involving categorical variables, the Pearson chi-square test was used when the expected value was greater than 5. If the expected value fell between 3 and 5, the chi-square Yates test was applied; Fisher’s exact test was used if the expected value was less than 3. For comparisons exceeding 2×2 categories, the Pearson chi-square test was used when the expected value exceeded 5, while the Fisher-Freeman-Halton test was used when the expected value was less than 5. The threshold for statistical significance was set at P < 0.05.

## Results

Twenty (11 males, 9 females; p = 1.00) of 23 initial patients completed the study. Two patients did not return for follow-up examinations, and one patient was excluded from the study because they did not follow the postsurgical oral hygiene instructions (Fig 1). The mean age of the participants was 32.55±6,16 (males, 32.27±5.83; females, 32.89±6.88). The nonsurgical periodontal treatments of the participants were completed within four months. The characteristics of treated teeth are shown in Table 1. There was no significant difference in teeth characteristics between the groups (p = 0.895).

**Table 1:** Demographic data of the participants.

	Groups		*p*
	OFD (n = 20)	OFD+ T-PRF (n = 20)	
***Gender***			
Male	11 (55%)	11 (55%)	1.00
Female	9 (45%)	9 (45%)	
***Teeth treated***			
Maxillary premolars	5	6	0.895
Maxillary molars	6	4	
Mandibular premolars	5	5	
Mandibular molars	4	5	

OFD: open flap debridement, T-PRF: Titanium-platelet-rich fibrin

### Clinical findings

Table 2 shows the mean differences in the clinical parameters from baseline to nine months. There was no significant difference between groups for PI (P = 0.570), mSBI (P = 0.562), or CAL gain (P = 0.118). However, there were significant differences in PPD reduction (P = 0.010), GML (P <0.001), and clinical endpoints (P = 0.038) between the groups from baseline to the end of the nine-month follow-up period.

**Table 2:** Mean differences of clinical parameters from baseline to 9 months.

Parameters	OFD	OFD + T-PRF	*P*
PI	0.07 ± 0.06	0.06 ± 0.05	0.570
mSBI	0.06 ± 0.03	0.04 ± 0.15	0.562
PPD reduction	3.04 ± 0.69*	3.82 ± 1.09*	0.010
CAL gain	2.35 ± 0.73	3.01 ± 1.70	0.118
GML	-0.40 ± 0.08	0.14 ± 0.12*	<0.001
CE	11*	17*	0.038

The values are expressed as the mean ± standard deviation.

* indicates significant differences between the periodontal parameters at baseline and 9 months after surgery; *P* <0.05.

OFD: open flap debridement; T-PRF: Titanium-platelet-rich fibrin; PI: plaque index; mSBI: modified sulcus bleeding index; PPD: probing pocket depth; CAL: clinical attachment level; GML: gingival marginal level; CE: the number of sites that reach the clinical endpoint: "≤4 sites with PD≥5 mm

### Radiographic findings

Intra and intergroup comparisons of the differences in PBS and the bone-filling ratio are shown in Table 3. PBS increased significantly in both groups (OFD, *P* = 0.010; OFD+T-PRF, *P*<0.001). Moreover, when the bone-filling rate was compared between groups, the rate was significantly greater in the OFD+T-PRF group (P <0.001).

**Table 3:** Intra- and intergroup comparisons of the differences in periodontal bone support and the bone filling ratio according to radiographic images.

		OFD	OFD+T-PRF	*P*
PBS	Baseline	0.45±0.19	0.47±0.18	0.734
	9 months	0.64±0.29*	0.73±0.31*	0.349
	*p*	0,010	<0,001	
Bone filling ratio (Difference between baseline to 9 months)		0.11±0.04	0.27±0.11^†^	<0.001

* indicates a statistically significant difference between baseline and 9 months after surgery within the groups (*P* <0.05).

^†^ indicates a statistically significant difference between the groups (*P* <0.05).

OFD: open flap debridement; T-PRF: Titanium-platelet-rich fibrin; PBS: periodontal bone support

### Biochemical findings

**Fig 2:**
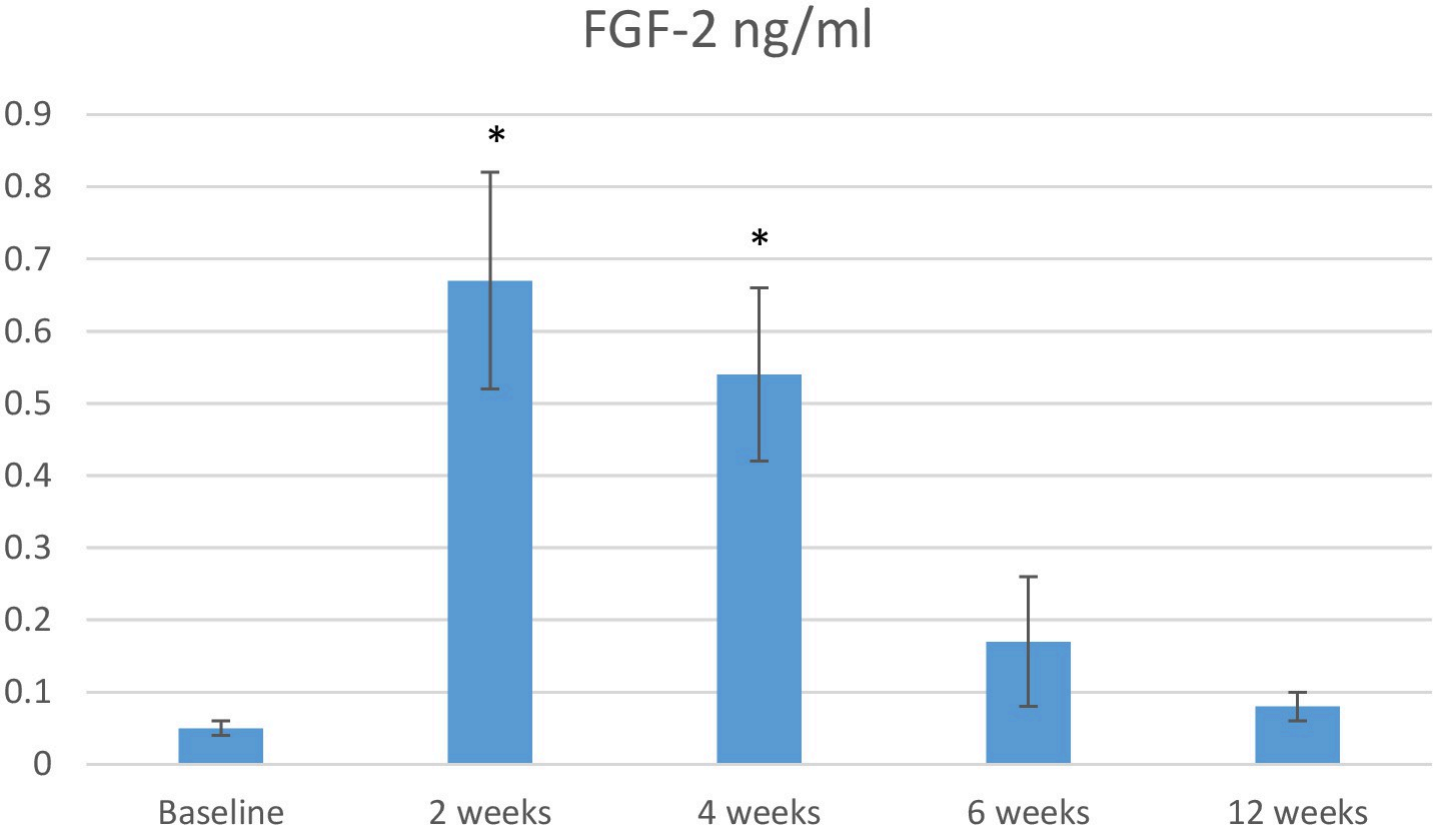
The mean difference in FGF-2 concentrations between groups at baseline and 12 weeks.

**Fig 3:**
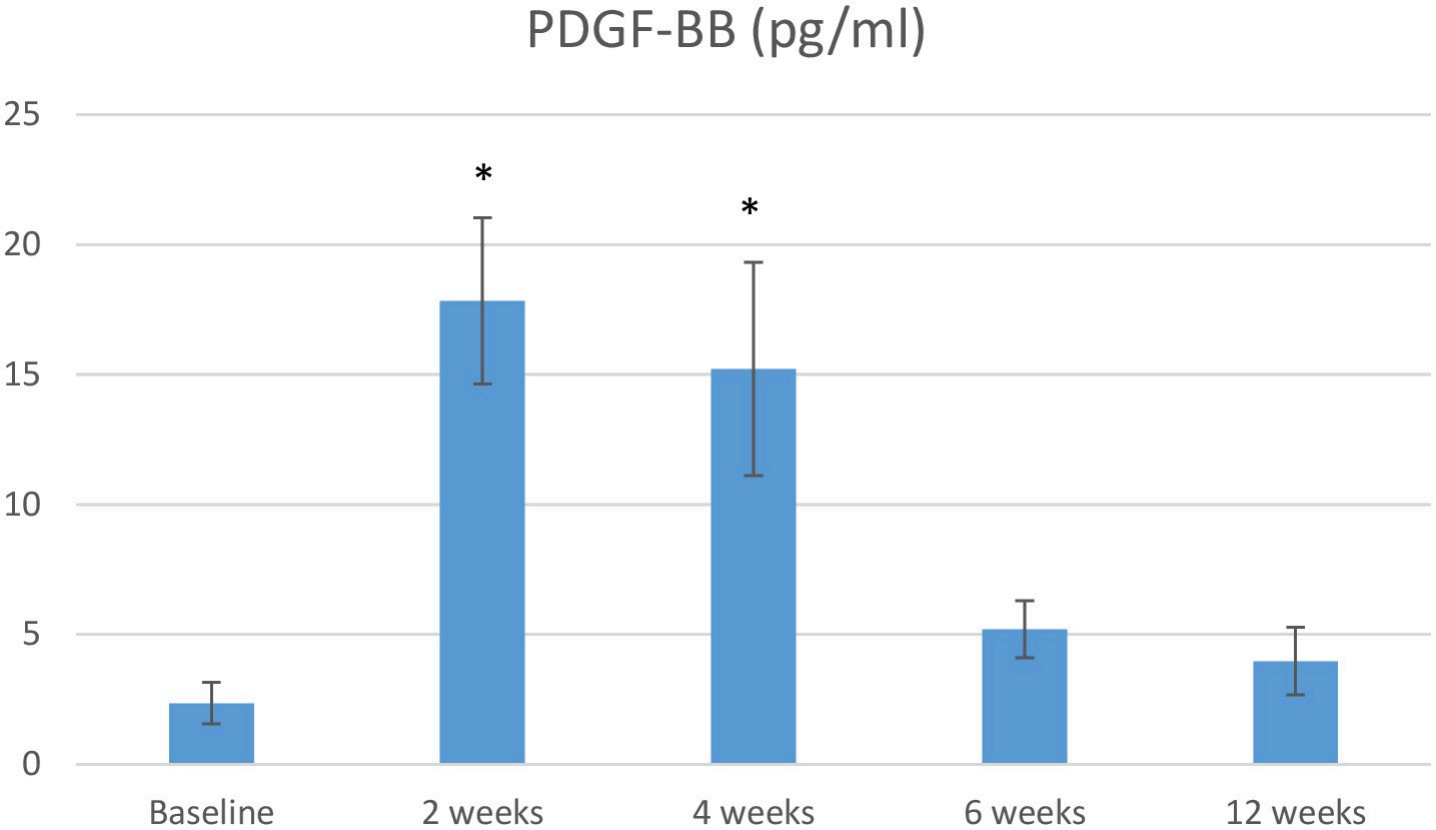
The mean difference in PDGF-BB concentrations between groups at baseline and 12 weeks.

**Fig 4:**
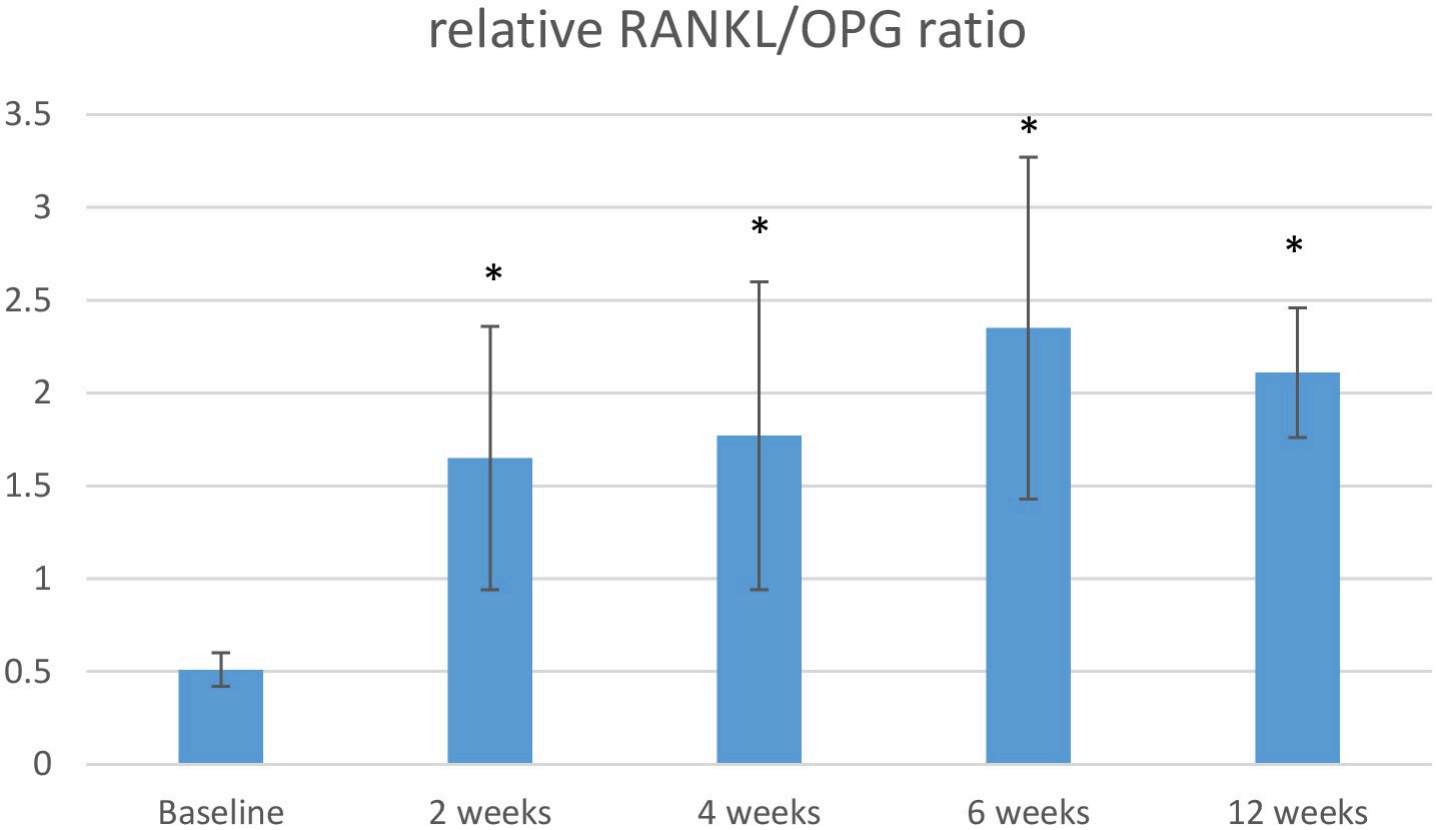
The mean difference in the relative RANKL/OPG ratio between groups at baseline and 12 weeks.

Differences in the GCF concentrations of FGF-2 and PDGF-BB over time between the OFD and OFD+T-PRF groups are shown in Figs 2 and 3, while differences in the RANKL/OPG ratio between the groups are shown in Fig 4.

The FGF-2 and PDGF-BB concentrations were greater in the OFD+T-PRF group. However, the increases in these parameters in comparison to those in the OFD group were significant only in the second (FGF-2, 0.67±0.15; PDGF-BB, 17.84±3,2) and fourth (FGF-2, 0.54±0.12; PDGF-BB, 15.22±4,1) weeks (*P* <0.05) of follow-up, respectively (Fig 2a and 2b). The relative RANKL/OPG ratio was calculated for each sample separately at baseline and and the second, fourth, sixth, and twelfth weeks. The differences between groups were significant in the second (1.65±0.71), fourth (1.77±0.83), sixth (2.35±0.92), and twelfth (2.11±0.35) weeks (*P* < 0.05) (Fig 2c).

## Discussion

Periodontal treatment aims to prevent the progression of periodontal infection and regenerate lost periodontal tissues [34]. According to previous studies, conventional periodontal treatment, such as OFD, is effective but inadequate at repairing periodontal IBDs and slowing the progression of periodontal disease. Moreover, treating periodontal bone defects with OFDs provides limited access to periodontal soft and hard tissue [35].

Recent studies have reported that using growth factor-containing biological materials in surgical procedures is advantageous. These biomaterials improve early wound healing, bone graft maturation, and the final aesthetic outcome of periodontal soft tissues around an implant and tooth [36, 37]. Compared to glass tubes, PRF obtained from titanium tubes has better biocompatibility and releases growth factors for a longer period due to its thicker and more polymerized structure. T-PRF is also preferred due to the fact that it is a cost-free material that only requires the blood of the patient [12]. Therefore, this study aimed to evaluate the efficacy of autologous T-PRF in the treatment of periodontal bone defects in a randomized controlled clinical trial [14].

In the past, many research studies have used bone grafts and membranes together [38] and PRF alone or in combination with grafts [39] in the regenerative periodontal treatment. Previous reports have been controversial regarding the use of platelet concentrates alone or in combination with bone graft substitutes. While some investigators concluded superior clinical efficacy in favor of combined use [40], others demonstrated no additional benefit [41]. In the present study, we used T-PRF alone as the graft and membrane material in the test sites.

The morphology of the tooth/root and the topography of the defect play an essential role in the success of a regenerative treatment. Molars are not preferred due to difficulty in surgical access and furcation involvement [32]. In the present study, there were no statistical difference of characteristics of treated teeth as a result of standardization. (p = 0.895) In regenerative treatments, the design of the flap is also important [42]. Several flap designs have been the subject of evaluation in regenerative procedures [43]. The conclusion of Schincaglia et al. [44] was that there were no significant differences between the single flap and double flap approaches. In our study, we performed full thickness mucoperiosteal double flap approach.

Reducing probing depths and increasing attachment levels are important clinical parameters that indicate successful periodontal regeneration therapy [37]. Results from the present study indicate that decreases in the PPD nine months following therapy compared with baseline were significantly greater (p = 0.010) in patients who underwent the OFD procedure combined with T-PRF; similar to studies by Thorat et al. [45] and Ustaoğlu et al. [32]. In a related study, Sharma et al. [8] reported that, after nine months of follow-up, OFD-treated regions achieved similar CALs as PRF- or PRP-treated regions. In this study, the difference in CAL gain was not significant (p = 0.118). Similar studies by Arabaci et al. [46], Chatterjee et al. [34], and Reddy et al. [47] showed a significant gain in clinical attachment level that differed from our results. However, a decrease in probing depth was similar to what we found, and new bone formation was greater when T-PRF was used with OFD procedures. Pradeep et al. [39] reported similar bone fill in 3-wall IBDs treated with PRF or PRP combined with OFD.

The differences in clinical and radiographic parameters between groups are mostly consistent with the findings of a systematic review that concluded that using specific biomaterials/biologicals is more efficient than OFDs for treating IBDs [4]. Our results support the positive contribution of T-PRF to periodontal wound healing in 3-walled IBDs.

In the present study, the concentrations of growth factors were significantly greater at T-PRF sites than at OFD sites two and four weeks after surgery, confirming the results of previous studies [46, 48]. Compared to PRF, T-PRF has a thicker, more prominent, and more cross-mesh structure, and its resorption time has been reported to be 30 days [49]. Therefore, T-PRF resorbs later in the flap than traditional PRF, and there is a longer release of growth factors. The high PDGF-BB and FGF-2 levels at the end of the fourth week in the present study are important findings that can be attributed to this situation. The release of growth factors stimulates the migration of tissue-forming cells and enhances wound healing and improved periodontal regeneration in T-PRF treated sites [50].

The molecular regulators of bone metabolism, OPG and RANKL, were also evaluated. The RANKL/OPG ratio in the OFD+T-PRF group was significantly lower than in the OFD group, indicating greater catabolic activity in the T-PRF-treated sites, consistent with the results from a similar study [46]. In addition, Tang et al. [51] reported that osteogenic induction significantly downregulated the RANKL/OPG ratio. In line with these results, the use of T-PRF in periodontal regeneration studies has shown positive effects, and this hypothesis was supported in this study.

Variations between the results of the present study and several studies in the literature may be explained by methodological differences such as tooth inclusion, defect morphology, regenerative treatment strategy, or the length of follow-up [32].

### Limitations of the study

Although our sample size was determined by reviewing the available literature, further clinical studies with larger sample sizes should be conducted to better determine the clinical and biochemical benefits of T-PRF. One limitation of our study could have arisen from the evaluation stage of the clinical and radiographic parameters. Evaluations made by multiple periodontists could have yielded more objective results. Although we standardized how we took radiographic images, differences may have arisen while recording the images, which could have affected the radiographic evaluation. We believe further studies using three-dimensional radiographs, which allow clearer information to be obtained for evaluating bone regeneration, will support our findings. In addition, clinical and radiographic periodontal examinations included in the study were recorded at the beginning and end of nine months. Further studies, where control measurements are made for six months post-surgery (a critical period in periodontal treatment), would be useful in evaluating the effectiveness of T-PRF for early recovery.

## Conclusions

To the best of our knowledge, this study is the first prospective, randomized, controlled clinical trial that investigates T-PRF in specific defect morphology type as 3-wall intrabone defects. According to results of recent study, a treatment approach using T-PRF has led to significant improvement in both clinical, radiographic, and biochemical parameters than OFD alone in the treatment of IBDs. Considering the statistically insignificant values between the groups, this may have been due to the sample size. This study has significant potential to lead scientific studies showing the possible use of T-PRF in the treatment of 3-wall intrabone defects.

## Supporting information

S1 File Receipt_clinicaltrials. (PDF)

S2 File CONSORT checklist. (PDF)

S1 Data (XLSX)
