# Supplementary material for: The effect of titanium-platelet rich fibrin on periodontal intrabony defects: A randomized controlled split-mouth clinical study
Source: PLoS One. 2024 Jun 6;19(6):e0304970. doi: 10.1371/journal.pone.0304970 (PMC11156295; doi:10.1371/journal.pone.0304970)
Supplement: S1 File — (PDF) [file pone.0304970.s001.pdf]

ClinicalTrials.gov PRS DRAFT Receipt (Working Version)
Last Update: 01/08/2024 16:30

ClinicalTrials.gov ID: NCT05409495

Study Identification

Unique Protocol ID: B.30.2.ATA.0.01.00/93

Brief Title: Titanium-prepared Platelet-rich Fibrin in Periodontal Regeneration

Official Title: The Effect of Titanium- Platelet Rich Fibrin in Periodontal Intrabony Defects: A Randomized Controlled Split-Mouth Clinical Study

Secondary IDs:

Study Status

Record Verification: June 2022

Overall Status: Completed

Study Start: April 1, 2021 [Actual]

Primary Completion: February 1, 2022 [Actual]

Study Completion: February 15, 2022 [Actual]

Sponsor/Collaborators

Sponsor: Ataturk University

Responsible Party: Principal Investigator
Investigator: Gurbet Alev OZTAS SAHINER [goztassahiner]
Official Title: Principal Investigator
Affiliation: Ataturk University

Collaborators:

Oversight

U.S. FDA-regulated Drug: No

U.S. FDA-regulated Device: No

U.S. FDA IND/IDE: No

Human Subjects Review: Board Status: Approved
Approval Number: B.30.2.ATA.0.01.00/93
Board Name: Atatürk University
Board Affiliation: Ethics Committee
Phone: +90 442 344 65 11
Email: atatipetikkurul@gmail.com
Address:

Ataturk University Faculty of Medicine Dean's Office/ Erzurum/ TURKEY

Data Monitoring: No

FDA Regulated Intervention: No

Study Description

Brief Summary: In this study, the possible effect of blood group distribution on the content of blood biomaterial was investigated. 64 volunteers were included in the study. Various parameters were evaluated. As a result, it was concluded that blood group distribution does not affect blood biomaterial content.

Detailed Description: The aim of this split-mouth, randomized, and controlled study was to compare open flap debridement (OFD) alone against OFD with autogenous Titanium-prepared platelet-rich fibrin (OFD+ T-PRF) combined in treating intrabony defects (IBD). Subjects were 20 systemically healthy patients with chronic periodontitis according to 2017 World Workshop. Bilateral operation sites in patients (40 sites) were randomly selected for OFD alone or OFD+ T-PRF combined. Clinical parameters (probing depth (PD), relative attachment level (RAL), and gingival marginal level(GML)), radiographic parameters (intrabone defects (IBDs) and periodontal bone support (PBS)), and growth factors levels (GFL) in gingival crevicular fluid (GCF) (platelet-derived growth factors (PDGF-BB), fibroblast growth factors (FGF-2), relative ratio of receptor activator nuclear factor kappa-B (RANKL)/osteoprotegerin (OPG)) were analyzed. The Wilcoxon signed-rank test, the Student's t-test, the two way ANOVA, and the Tukey post hoc test were used for statistical analysis.

Conditions

Conditions: Platelet-Rich Fibrin

Keywords: chronic periodontitis
growth factors
guided tissue regeneration
periodontal
platelet-derived growth factor
platelet-rich fibrin

Study Design

Study Type: Interventional

Primary Purpose: Treatment

Study Phase: N/A

Interventional Study Model: Parallel Assignment
Two different treatment modalities to treat deep periodontal intrabony defects were compared in this split-mouth, randomized, parallel and clinical study. The control group defects were treated with OFD only while the test group defects were treated with OFD supplemented with T-PRF. The same periodontal treatment procedure was applied in both groups, except for the use of T-PRF. Clinical and radiographic parameters were measured from baseline to 9 months after surgery.

Number of Arms: 2

Masking: None (Open Label)

Allocation: Randomized

Enrollment: 20 [Actual]

Arms and Interventions

Arms	Assigned Interventions
Experimental: The control group treated with open flap debridement (OFD)<br>The control group periodontal intrabony defects were treated with open flap debridement (OFD) only.	Procedure/Surgery: periodontal surgical procedure (open flap debridement)<br>All surgical procedures were performed by the second periodontist. 0.12% Chlorhexidine digluconate (CHX) rinse for intraoral antisepsis and a povidone iodine solution was used for extraoral antisepsis. After local anesthesia (2% lidocaine with epinephrine 1:100,000/ Astra, Westbrough, MA) was applied, the full thickness trapezoidal flap was raised large enough to provide adequate view of the defect area. Subgingival debridement and root planning were performed with the use of area-specific curets (Gracey curets, Hu-Friedy), and granulation tissue was removed The IBD area in the control group was closed without applying any material. Then mucoperiosteal flaps were repositioned with sutured with 4/0 monoprolene sutures.
Experimental: The test group treated with OFD +autogenous Titanium-prepared platelet-rich fibrin (OFD+ T-PRF)<br>The test group periodontal intrabony defects were treated with open flap debridement (OFD) with autogenous Titanium-prepared platelet-rich fibrin (OFD+ T-PRF) combined.	Procedure/Surgery: periodontal surgical procedure (OFD +autogenous Titanium-prepared platelet-rich fibrin (OFD+ T-PRF))<br>All surgical procedures were performed by the second periodontist. 0.12% Chlorhexidine digluconate (CHX) rinse for intraoral antisepsis and a povidone iodine solution was used for extraoral antisepsis. After local anesthesia (2% lidocaine with epinephrine 1:100,000/ Astra, Westbrough, MA) was applied, the full thickness trapezoidal flap was raised large enough to provide adequate view of the defect area. Subgingival debridement and root planning were performed with the use of area-specific curets (Gracey curets, Hu-Friedy), and granulation tissue was removed (Figure 2a). The blood supply of the defect areas was taken into account. At the test site, IBDs were filled with T-PRF and T-PRF membranes were adapted over the defects both buccally and lingually, in addition to OFD (Figure 2b). Then mucoperiosteal flaps were repositioned with sutured with 4/0 monoprolene sutures.

Outcome Measures

Primary Outcome Measure:

1. site-specific plaque index (PI) (Silness & Loe)
   measurement of plaque accumulated on the tooth surface

   [Time Frame: 9 month]

2. modified sulcus bleeding index (mSBI)
   assessment of bleeding gums

   [Time Frame: 9 month]

3. probing depth from the gingival margin (PD)
   evaluated from the gingival margin to the base of the pocket

[Time Frame: 9 month]

4. gingival marginal level (GML)
   measured from the apical most end of the stent to the crest of the gingival margin

   [Time Frame: 9 month]

5. relative attachment level (RAL)
   evaluated from the cementoenamel junction to the base of the pocket and gingival marginal level

   [Time Frame: 9 month]

Secondary Outcome Measure:

6. fibroblast growth factors (FGF-2)
   growth factor affecting periodontal regeneration

   [Time Frame: 12 weeks]

7. platelet-derived growth factors (PDGF-BB)
   growth factor affecting periodontal regeneration

   [Time Frame: 12 weeks]

8. Gingival Crevicular Fluid Collection; The gingival crevicular fluid (GCF) sample was collected to biochemically evaluate the patient's periodontal tissue healing.
   relative ratio of receptor activator nuclear factor kappa-B (RANKL)/osteoprotegerin (OPG))

   [Time Frame: 12 weeks]

Other Pre-specified Outcome Measures:

9. While examining the radiographic intraosseous defect, the distance between the alveolar bone crest and the base of the defect was taken into account. This distance (IBD) was evaluated using computer aided software.
   Radiographic Measurements; Measurements were made on radiographic images in order to evaluate the healing of the patient's bone tissue.

   [Time Frame: 9 month]

10. Also while measuring periodontal bone support (PBS) using radiographic images used Image Tool v.3.0 (UTHSCSA).
    Radiographic Measurements; Measurements were made on radiographic images in order to evaluate the healing of the patient's bone tissue.

    [Time Frame: 9 month]

Eligibility

Minimum Age:	20 Years
Maximum Age:	60 Years
Sex:	All
Gender Based:	No
Accepts Healthy Volunteers:	Yes
Criteria:	Inclusion Criteria:

1)Patients with bilaterally similar periodontal intrabone defects (IBDs)

Exclusion Criteria:

1. Who did not show the necessary oral hygiene during the non-surgical periodontal treatment process,
2. history of periodontal therapy in the preceding 1 year,

3. presence of devital tooth, Grade II, or higher mobility of the tooth, and less than 3 bone walls or a defect in the furcation at the site of the bone defect,
4. history of any systemic diseases that can alter the course of the periodontal disease,
5. smokers,
6. use of antibiotics,
7. pregnant/lactating women.

Contacts/Locations

Central Contact Person: Gurbet A Oztas Sahiner, Dr
Telephone: 905063279811
Email: alev.oztas@atauni.edu.tr

Central Contact Backup:

Study Officials: Didem Ozkal Eminoglu, Dr
Study Director
Atatürk University Faculty of Dentistry Department of Periodontology

Locations: **Turkey**
Atatürk University Faculty of Dentistry Department of Peirodontology
Erzurum, Turkey, 25240
Contact: Gurbet A Oztas Sahiner, Dr 905063279811
alev.oztas@atauni.edu.tr

IPDSharing

Plan to Share IPD: Yes
If the data of the study is requested by other researchers, the study supervisor can be contacted.

Supporting Information:
Study Protocol
Statistical Analysis Plan (SAP)
Informed Consent Form (ICF)
Clinical Study Report (CSR)
Analytic Code

Time Frame:
There is no specific time restriction for this.

Access Criteria:
There is no specific time criterion for this.

URL: http://alev.oztas@atauni.edu.tr

References

Citations: **[Study Results]** Cochran DL, Wozney JM. Biological mediators for periodontal regeneration. Periodontol 2000. 1999 Feb;19:40-58. doi: 10.1111/j.1600-0757.1999.tb00146.x. PubMed 10321215

[Study Results] Dangaria SJ, Ito Y, Walker C, Druzinsky R, Luan X, Diekwisch TG. Extracellular matrix-mediated differentiation of periodontal progenitor cells. Differentiation. 2009 Sep-Oct;78(2-3):79-90. doi: 10.1016/j.diff.2009.03.005. Epub 2009 May 9. PubMed 19433344

[Study Results] Whitman DH, Berry RL, Green DM. Platelet gel: an autologous alternative to fibrin glue with applications in oral and maxillofacial surgery. J Oral Maxillofac Surg. 1997 Nov;55(11):1294-9. doi: 10.1016/s0278-2391(97)90187-7. PubMed 9371122

[Study Results] Dohan Ehrenfest DM, Rasmusson L, Albrektsson T. Classification of platelet concentrates: from pure platelet-rich plasma (P-PRP) to leucocyte- and platelet-rich fibrin (L-PRF). Trends Biotechnol. 2009 Mar;27(3):158-67. doi: 10.1016/j.tibtech.2008.11.009. Epub 2009 Jan 31. PubMed 19187989

[Study Results] Dohan DM, Choukroun J, Diss A, Dohan SL, Dohan AJ, Mouhyi J, Gogly B. Platelet-rich fibrin (PRF): a second-generation platelet concentrate. Part II: platelet-related biologic features. Oral Surg Oral Med Oral Pathol Oral Radiol Endod. 2006 Mar;101(3):e45-50. doi: 10.1016/j.tripleo.2005.07.009. Epub 2006 Jan 10. PubMed 16504850

[Study Results] Kang YH, Jeon SH, Park JY, Chung JH, Choung YH, Choung HW, Kim ES, Choung PH. Platelet-rich fibrin is a Bioscaffold and reservoir of growth factors for tissue regeneration. Tissue Eng Part A. 2011 Feb;17(3-4):349-59. doi: 10.1089/ten.TEA.2010.0327. Epub 2010 Dec 31. PubMed 20799908

[Study Results] Bussel JB, Kunicki TJ, Michelson AD. Platelets: New Understanding of Platelet Glycoproteins and Their Role in Disease. Hematology Am Soc Hematol Educ Program. 2000:222-240. doi: 10.1182/asheducation-2000.1.222. PubMed 11701544

[Study Results] Barbalic M, Dupuis J, Dehghan A, Bis JC, Hoogeveen RC, Schnabel RB, Nambi V, Bretler M, Smith NL, Peters A, Lu C, Tracy RP, Aleksic N, Heeriga J, Keaney JF Jr, Rice K, Lip GY, Vasan RS, Glazer NL, Larson MG, Uitterlinden AG, Yamamoto J, Durda P, Haritunians T, Psaty BM, Boerwinkle E, Hofman A, Koenig W, Jenny NS, Witteman JC, Ballantyne C, Benjamin EJ. Large-scale genomic studies reveal central role of ABO in sP-selectin and sICAM-1 levels. Hum Mol Genet. 2010 May 1;19(9):1863-72. doi: 10.1093/hmg/ddq061. Epub 2010 Feb 18. PubMed 20167578

[Study Results] Mohanty D, Ghosh K, Marwaha N, Kaur S, Chauhan AP, Das KC. Major blood group antigens--a determinant of factor VIII levels in blood? Thromb Haemost. 1984 Jul 29;51(3):414. No abstract available. PubMed 6437009

[Study Results] Ghanaati S, Booms P, Orlowska A, Kubesch A, Lorenz J, Rutkowski J, Landes C, Sader R, Kirkpatrick C, Choukroun J. Advanced platelet-rich fibrin: a new concept for cell-based tissue engineering by means of inflammatory cells. J Oral Implantol. 2014 Dec;40(6):679-89. doi: 10.1563/aaid-joi-D-14-00138. PubMed 24945603

[Study Results] Chatterjee A, Pradeep AR, Garg V, Yajamanya S, Ali MM, Priya VS. Treatment of periodontal intrabony defects using autologous platelet-rich fibrin and titanium platelet-rich fibrin: a randomized, clinical, comparative study. J Investig Clin Dent. 2017 Aug;8(3). doi: 10.1111/jicd.12231. Epub 2016 Jul 31. PubMed 27477110

[Study Results] Kim TH, Kim SH, Sandor GK, Kim YD. Comparison of platelet-rich plasma (PRP), platelet-rich fibrin (PRF), and concentrated growth factor (CGF) in rabbit-skull defect healing. Arch Oral Biol. 2014 May;59(5):550-8. doi: 10.1016/j.archoralbio.2014.02.004. Epub 2014 Feb 15. PubMed 24667430

[Study Results] Kobayashi E, Fluckiger L, Fujioka-Kobayashi M, Sawada K, Sculean A, Schaller B, Miron RJ. Comparative release of growth factors from PRP, PRF, and advanced-PRF. Clin Oral Investig. 2016 Dec;20(9):2353-2360. doi: 10.1007/s00784-016-1719-1. Epub 2016 Jan 25. PubMed 26809431

[Study Results] Kumar RV, Shubhashini N. Platelet rich fibrin: a new paradigm in periodontal regeneration. Cell Tissue Bank. 2013 Sep;14(3):453-63. doi: 10.1007/s10561-012-9349-6. Epub 2012 Nov 11. PubMed 23143637

[Study Results] Masuki H, Okudera T, Watanebe T, Suzuki M, Nishiyama K, Okudera H, Nakata K, Uematsu K, Su CY, Kawase T. Growth factor and pro-inflammatory cytokine contents in platelet-rich plasma (PRP), plasma rich in growth factors (PRGF), advanced platelet-rich fibrin (A-PRF), and concentrated growth factors (CGF). Int J Implant Dent. 2016 Dec;2(1):19. doi: 10.1186/s40729-016-0052-4. Epub 2016 Aug 22. PubMed 27747711

[Study Results] Dohan Ehrenfest DM, Bielecki T, Jimbo R, Barbe G, Del Corso M, Inchingolo F, Sammartino G. Do the fibrin architecture and leukocyte content influence the growth factor release of platelet concentrates? An evidence-based answer comparing a pure platelet-rich plasma (P-PRP) gel and a leukocyte- and platelet-rich fibrin (L-PRF). Curr Pharm Biotechnol. 2012 Jun;13(7):1145-52. doi: 10.2174/138920112800624382. PubMed 21740377

[Study Results] Su CY, Kuo YP, Tseng YH, Su CH, Burnouf T. In vitro release of growth factors from platelet-rich fibrin (PRF): a proposal to optimize the clinical applications of PRF. Oral Surg Oral Med Oral Pathol Oral Radiol Endod. 2009 Jul;108(1):56-61. doi: 10.1016/j.tripleo.2009.02.004. Epub 2009 May 17. PubMed 19451002

[Study Results] Clipet F, Tricot S, Alno N, Massot M, Solhi H, Cathelineau G, Perez F, De Mello G, Pellen-Mussi P. In vitro effects of Choukroun's platelet-rich fibrin conditioned medium on 3 different cell lines implicated in dental implantology. Implant Dent. 2012 Feb;21(1):51-6. doi: 10.1097/ID.0b013e31822b9cb4. PubMed 21986450

[Study Results] Dereka XE, Markopoulou CE, Vrotsos IA. Role of growth factors on periodontal repair. Growth Factors. 2006 Dec;24(4):260-7. doi: 10.1080/08977190601060990. PubMed 17381067

[Study Results] Selezneva IA, Gilmiyarova FN, Borodina IA, Ereshchenko AA, Gilmiyarov EM, Kartashov VV. [capital ES, Cyrilliclinicmolecular indicators of inflammatory destructive damage of the oral cavity in periodontitis in persons with various group accessories of blood.]. Klin Lab Diagn. 2020;65(2):100-105. doi: 10.18821/0869-2084-2020-65-2-100-105. Russian. PubMed 32159307

[Study Results] Arabaci T, Albayrak M. Titanium-prepared platelet-rich fibrin provides advantages on periodontal healing: A randomized split-mouth clinical study. J Periodontol. 2018 Mar;89(3):255-264. doi: 10.1002/JPER.17-0294. PubMed 29543995

[Study Results] Dohan Ehrenfest DM, Pinto NR, Pereda A, Jimenez P, Corso MD, Kang BS, Nally M, Lanata N, Wang HL, Quirynen M. The impact of the centrifuge characteristics and centrifugation protocols on the cells, growth factors, and fibrin architecture of a leukocyte- and platelet-rich fibrin (L-PRF) clot and membrane. Platelets. 2018 Mar;29(2):171-184. doi: 10.1080/09537104.2017.1293812. Epub 2017 Apr 24. PubMed 28437133

[Study Results] Choukroun J, Ghanaati S. Reduction of relative centrifugation force within injectable platelet-rich-fibrin (PRF) concentrates advances patients' own inflammatory cells, platelets and growth factors: the first introduction to the low speed centrifugation concept. Eur J Trauma Emerg Surg. 2018 Feb;44(1):87-95. doi: 10.1007/s00068-017-0767-9. Epub 2017 Mar 10. PubMed 28283682

Links:

Available IPD/Information:

U.S. National Library of Medicine | U.S. National Institutes of Health | U.S. Department of Health & Human Services
